# Daily consumption of one teaspoon of trehalose can help maintain glucose homeostasis: a double-blind, randomized controlled trial conducted in healthy volunteers

**DOI:** 10.1186/s12937-020-00586-0

**Published:** 2020-07-09

**Authors:** Chiyo Yoshizane, Akiko Mizote, Chikako Arai, Norie Arai, Rieko Ogawa, Shin Endo, Hitoshi Mitsuzumi, Shimpei Ushio

**Affiliations:** Hayashibara Co. Ltd., 675 Fujisaki, Naka-ku, Okayama, 702-8006 Japan

**Keywords:** Trehalose, Glucose tolerance, Insulin resistance, Postprandial blood glucose, Two-hour plasma glucose

## Abstract

**Background:**

Trehalose is a natural disaccharide that is widely distributed. A previous study has shown that daily consumption of 10 g of trehalose improves glucose tolerance in individuals with signs of metabolic syndrome. In the present study, we determined whether a lower dose (3.3 g/day) of trehalose improves glucose tolerance in healthy Japanese volunteers.

**Methods:**

This was a randomized, double-blind, placebo-controlled study of healthy Japanese participants (*n* = 50). Each consumed 3.3 g of trehalose (*n* = 25) or sucrose (*n* = 25) daily for 78 days. Their body compositions were assessed following 0, 4, 8, and 12 weeks; and serum biochemical parameters were assayed and oral 75-g glucose tolerance tests were performed at baseline and after 12 weeks.

**Results:**

There were similar changes in body composition and serum biochemistry consistent with established seasonal variations in both groups, but there were no differences in any of these parameters between the two groups. However, whereas after 12 weeks of sucrose consumption, the plasma glucose concentration 2 h after a 75-g glucose load was significantly higher than the fasting concentration, after 12 weeks of trehalose consumption the fasting and 2-h plasma glucose concentrations were similar. Furthermore, an analysis of the participants with relatively high postprandial blood glucose showed that the plasma glucose concentration 2 h after a 75-g glucose load was significantly lower in the trehalose group than in the sucrose group.

**Conclusions:**

Our findings suggest that trehalose helps lower postprandial blood glucose in healthy humans with higher postprandial glucose levels within the normal range, and may therefore contribute to the prevention of pathologies that are predisposed to by postprandial hyperglycemia,, even if the daily intake of trehalose is only 3.3 g, an amount that is easily incorporated into a meal.

**Trial registration:**

UMIN, UMIN000033536. Registered 27 July 2018.

## Background

Trehalose is a non-reducing disaccharide composed of two α-glucose molecules that are linked by α 1,1-glycosidic bond. This saccharide is digested by the enzyme trehalase in the intestine, liberating glucose, which is absorbed. Trehalose is widely distributed, being found in beans, seaweeds, mushrooms, and yeasts, and has therefore been consumed for millennia [1, 2]. In addition, it has low sweetness, a clean finish, and excellent physical properties, including an anti-aging effect on starch and protein stabilization [1].

In the last 20 years, trehalose has been found to be useful for the prevention of a number of common health problems, including osteoporosis [3], metabolic syndrome [4, 5], and Alzheimer’s disease [6]. With relevance to metabolic syndrome, we have previously reported that trehalose suppresses visceral adipocyte hypertrophy and ameliorates insulin resistance in mice fed a high-fat diet (HFD) [4, 5]. Furthermore, we have demonstrated that daily consumption of 10 g of trehalose improves glucose tolerance in healthy humans [7], evaluated by oral glucose tolerance testing (OGTT). High postprandial blood glucose concentrations are associated with a higher risk of arteriosclerosis [8, 9]. Specifically, high 2-h plasma glucose concentrations during OGTT (2-h PG) have been shown to be a reliable predictor of incident coronary heart disease and cardiovascular mortality [10] in cohort studies, such as the DECODE [11] and DECODA [12] studies.

In previous studies, we have shown that daily consumption of 2.5 and 0.3% (weight/volume; w/v) trehalose reduces adipocyte hypertrophy and ameliorates insulin resistance in HFD-fed mice [4, 5]. A daily intake of 2.5% (w/v) trehalose is equivalent to 1.6 g/kg body mass/day in mice and 10 g/day in a person weighing 60 kg according to the FDA guidelines [13]. Furthermore, we recently showed that mesenteric adipocyte hypertrophy is reduced, even when only 0.1% (w/v) trehalose is consumed by HFD-fed mice (unpublished). Therefore, even in humans, the consumption of even smaller amount of trehalose might be able to ameliorate glucose tolerance by reducing adipocyte hypertrophy.

If trehalose were effective at improving glucose tolerance at low doses, it could be included in various foods to provide health benefits. Therefore, the purpose of the present study was to determine the reproducibility of the improvement in glucose tolerance induced by trehalose consumption and whether the same effect is induced by the regular consumption of a small amount of trehalose by healthy humans.

## Methods

### Test substances

TREHA™ (Hayashibara Co. Ltd., Okayama, Japan) was used as the trehalose source in this study, in 1.9-g doses. The administered dose was equivalent to 1.65 g of anhydrous trehalose, because TREHA™ contains > 98.0% trehalose dihydrate. Extra-fine granulated sugar (Parl Ace Corp., Tokyo, Japan) was used as the sucrose source, in 1.65 g doses, and this served as the control substance for this study. The energy content of trehalose and sucrose is the same (16.7 kJ/g). These two substances were granulated to the same grain size and packed in identical plain silver film bags,. They were devised so that they could not be distinguished .

### Participants

Participants who were willing to participate in this study were evaluated by a medical doctor and were included in the study if they met the inclusion criteria;healthy Japanese adults, fasting blood glucose < 110 mg/ dL, employees at the Hayashibara CO.,LTD., subjects who can comply with the instruction of conducting the study. Participants were excluded in cases of a history of sever disorders, pregnant or lactation, a history of hyperglycemia. From the 51 subjects who applied for this study, 50 healthy adult Japanese participants (20 women and 30 men) were recruited according to criteria. Each was given a full explanation, both written and oral, regarding the purpose and procedure of the study, and written informed consent was obtained from each. The participants were instructed to make no further changes to their diet or lifestyle for the duration of the study.

### Study design

This study was designed as a randomized, double-blind, placebo-controlled, parallel- group trial. An individual who was not directly involved in the study randomly assigned the participants to two groups of 25, such that the groups had the same sex ratio and on the basis of their fasting blood glucose and 2-h PG values during OGTT. One group received trehalose and the other received sucrose. Double-blinding was ensured by the use of identical opaque sachets, outer packaging, labeling and color for both the compounds being administered. The identity of the substance being consumed by each participant remained confidential until all the data had been finalized, and the participants were blinded throughout the trial.

Each participant consumed one bag of substance twice a day for 78 days, such that the daily intake of the substances was approximately 3.3 g/day. The participants were permitted to prepare the substances for consumption in a variety of ways, such as by sprinkling them on meals or dissolving them in beverages. To evaluate the effects of each substance, body composition was assessed following 0, 4, 8, and 12 weeks of consumption. In addition, OGTT was performed at 0 and 12 weeks. This study was performed between August and December 2018.

### Ethical considerations

This study was performed under the supervision of a medical doctor and conducted in accordance with Hayashibara Co. Ltd. Ethics Committee Approval number 215, and was registered with the University Hospital Medical Information Network (UMIN) Center (UMIN000033536). The study was conducted in accordance with the principles of the Declaration of Helsinki (adopted in 1964 and revised in 2013) and the Japanese Ethical Guidelines for Medical and Health Research Involving Human Subjects (adopted in 2014 and revised in 2017).

### Body composition

The participants were prohibited from eating and drinking, except for a small amount of water, from 9 pm the day before their visit, until all the investigations carried out on the morning of the visit had been completed. Body mass, body fat%, fat mass, muscle mass, body water, and bone mass were measured using a body composition analyzer (MC-780A; Tanita Co., Ltd., Tokyo, Japan). The percentages of truncal fat and waist circumference were measured using an abdominal fat analyzer (AB-140; Tanita). Blood pressure was measured using blood pressure monitors (HEM-7020; Omron Healthcare Co., Ltd., Kyoto, Japan). Body Mass Index (BMI) was calculated by dividing body mass (kg) by height (m), squared.

### Plasma biochemistry and oral glucose tolerance testing

Blood samples were drawn after an overnight fast at baseline and after 12 weeks of test substance consumption. OGTT was performed after an overnight fast. Each participant was administered 75 g glucose in 200 g water and blood samples were collected before and 2 h after this glucose load. Plasma was obtained from the fasting blood samples to measure the fasting plasma glucose (FPG), insulin, HbA1c, total cholesterol, low-density lipoprotein (LDL)-cholesterol, high-density lipoprotein (HDL)-cholesterol, triglyceride (TG), total plasminogen activator-inhibitor-1(PAI-1), aspartate transaminase (AST), alanine transaminase (ALT), gamma-glutamyl transpeptidase (γ-GTP) and high-molecular weight (HMW) adiponectin concentrations. In addition, blood samples collected 2 h after glucose loading were used to measure 2-h PG and plasma insulin concentrations. These analyses were performed by the Okayama Medical Association Test Center (Okayama, Japan).

Homeostasis model assessment-insulin resistance (HOMA-IR) and homeostatic model assessment-beta cell function (HOMA-β) were calculated as follows: HOMA-IR = fasting glucose (mg/dL) × fasting insulin (μIU/mL) /405; HOMA-β = (fasting insulin (μIU/mL) × 360) / (fasting glucose (mg/dL) − 63).

During the intervention period, each participant recorded the ingestion of each test sample and any symptoms in a diary. In addition, before and after the test period, each completed a comprehensive questionnaire regarding their lifestyle and health, including a semi-quantitative food frequency questionnaire based on food group (FFQg), using Excel EiyokunTM v3.5 FFQg (Kenpakusha, Tokyo, Japan). FFQg is a food intake survey that evaluates the contents of a daily diet with the simple questions consisting of 29 food groups and 10 different cooking methods. This was conducted during week 0, before the intervention, and during the final week (week 12) of the intervention. The food consumed was analyzed during these periods to ensure that nutritional intake did not change significantly during the trial.

### Subset analysis

The data obtained from all the participants were analyzed in the first instance. However, we thought that it might not be possible to detect a lowering of plasma glucose concentrations in healthy people who did not originally have high postprandial blood glucose concentrations. Therefore, we also conducted a separate analysis of the participants who had relatively high postprandial blood glucose concentrations. Then, we selected 13 members of each group, whose percentage 2-h PG relative to FPG (2-h PG%) at baseline (week 0) exceeded the mean for all the participants and analyzed their data.

### Statistics

Data are shown as means ± standard deviations. Statistical analyses were performed using SPSS Statistics for Windows, Version 25 (IBM, Armonk, NY, USA). Comparisons of data between two groups in the same week were made using the Mann-Whitney U-test. Comparisons of data between week 0 and week 4, 8, and 12 values in the same group were made using the Wilcoxon signed-rank test. Statistical significance was accepted when *P* < 0.05. Spearman’s rank correlations were used to evaluate the relationships between 2-h PG and other clinical outcomes.

## Results

### Participation and baseline information

Table 1 shows the baseline characteristics of the participants. None of the participants withdrew from the trial during the study period because of adverse effects related to the test substances and there were no major deviations from the protocol. The intake rates of the test substances were 96.6 ± 3.9% overall, 96.6 ± 3.6% for the trehalose group, and 96.6 ± 4.3% for the sucrose group. The FFQg showed no differences between the two groups.

**Table 1:**
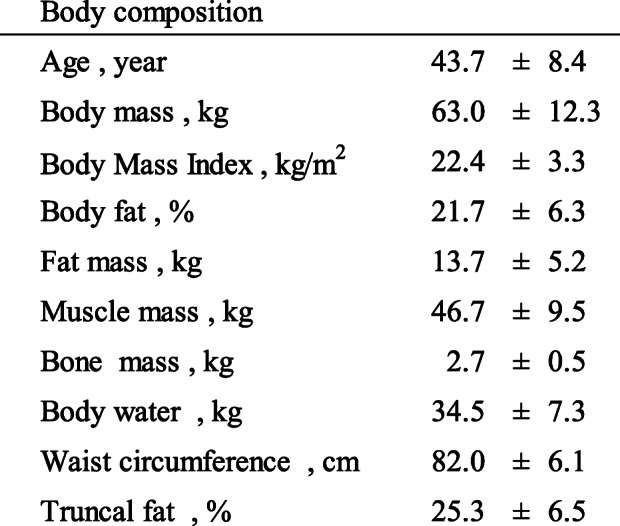
Participant characteristics at baseline

Data are expressed as mean ± SD (*n* = 50)

### Body composition and blood biochemistry

The results of physical examination and laboratory testing of plasma samples are shown in Table 2. There were no significant changes in the body mass of either group during the study period. However, body fat increased significantly in both groups during the study period, although the amount of truncal fat did not increase. In the sucrose group, waist circumference (WC) increased significantly. In addition, muscle mass, body water, and bone mass decreased significantly in both groups. However, there were no significant differences between the two groups with respect to any body composition parameter (Table 2).

**Table 2:**
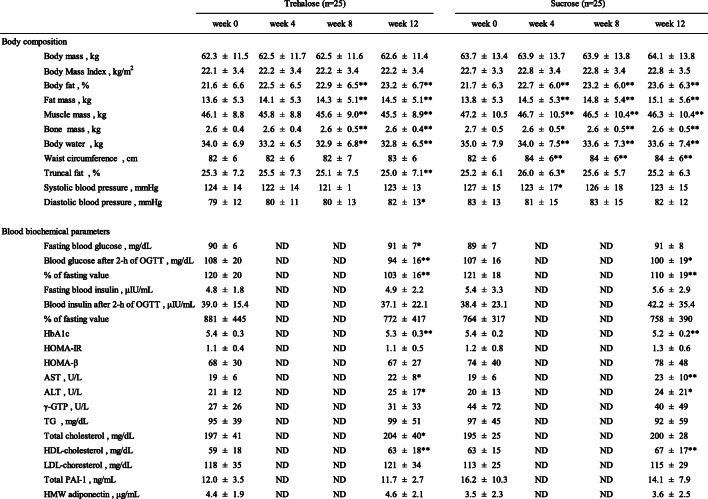
Body composition and blood biochemical parameters in the participants

*HOMA-IR* homeostasis model assessment-insulin resistance; *HOMA-β* homeostasis model assessment-beta cell function; *AST* aspartate transaminase; *ALT* alanine transaminase; *γ-GTP* gamma-glutamyl transpeptidase; *TG* triglyceride; *PAI-1* plasminogen activator-inhibitor-1; *HMW adiponectin* high-molecular weight adiponectin

Data are expressed as mean ± SD (*n* = 25). Comparisons between two groups in the same week were made using the Mann-Whitney U-test, and week 0 values and week 4, 8, and 12 values were compared using the Wilcoxon signed-rank test. *P* value: * < 0.05, ** < 0.01 vs. the week 0 value

**Fig. 1:**
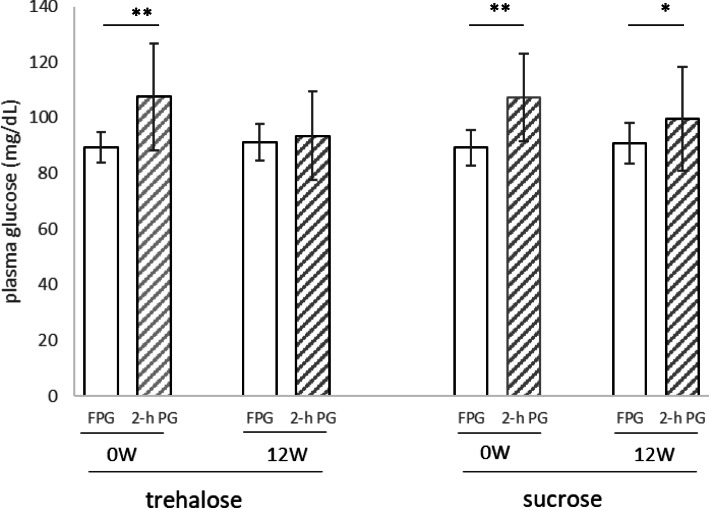
Fasting and 2-h plasma glucose (FPG, 2-h PG) after a 75-g glucose load in the complete group of participants. Left: trehalose intake group; right: sucrose intake group. Data are expressed as mean ± SD (*n* = 25). Comparisons of FPG and 2-h PG between the groups were made using the Wilcoxon signed-rank test. *P* values: *p < 0.05, **p < 0.01

Plasma AST and ALT activities and HDL-cholesterol concentration increased significantly during the test period in both groups. In contrast, no changes were observed in the TG, γ-GTP, PAI-1, or HMW adiponectin in either group. The 2-h PG and the ratio of 2-h PG to FPG (%) were significantly lower after the study period than before in both groups. However, there were no differences between the two groups with respect to any of these parameters (Table 2, Fig. 1).

The primary endpoint of this study was glucose tolerance, which is shown in Fig. 1 as a comparison of 2-h PG and FPG during OGTT. In both groups at the start of the study, 2-h PG was significantly higher than FPG. However, at the end of the test period, 2-h PG was significantly higher than FPG in the sucrose group, but there was no difference between these parameters in the trehalose group (Table 2, Fig. 1).

### 2-h PG values during OGTT in participants with high baseline postprandial blood glucose concentrations

**Fig. 2:**
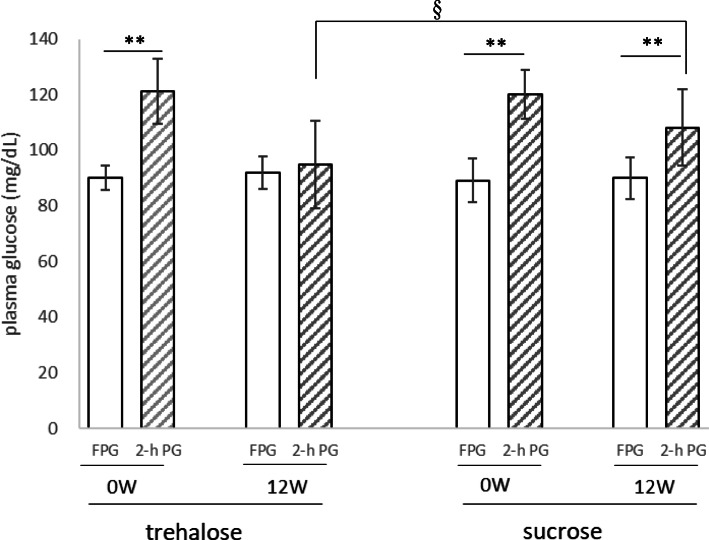
Fasting and 2-h plasma glucose (FPG, 2-h PG) after a 75-g glucose load in participants with a postprandial blood glucose concentration higher than the mean. Left: trehalose intake group; right: sucrose intake group. Data are expressed as mean ± SD (*n* = 13). Comparisons of FPG and 2-h PG between the groups were made using the Wilcoxon signed-rank test. *P* values: *p < 0.05, **p < 0.01. Comparisons between the trehalose and sucrose intake groups were made using the Mann-Whitney U-test. *P* value: ^§^*p* < 0.05

Data from the participants whose 2-h PG% values were higher than the mean value (120%) for all the participants were then analyzed (Table 3). In this subset of participants, after 12 weeks of trehalose or sucrose consumption, 2-h PG was significantly lower than at baseline, as for the complete set of participants. However, in the trehalose group, 2-h PG had returned to the level of FPG, but this did not occur in the sucrose group (Fig. 2). In addition, 2-h PG in the trehalose group was significantly lower than in the sucrose group (Table 3, Fig. 2).

**Table 3:**
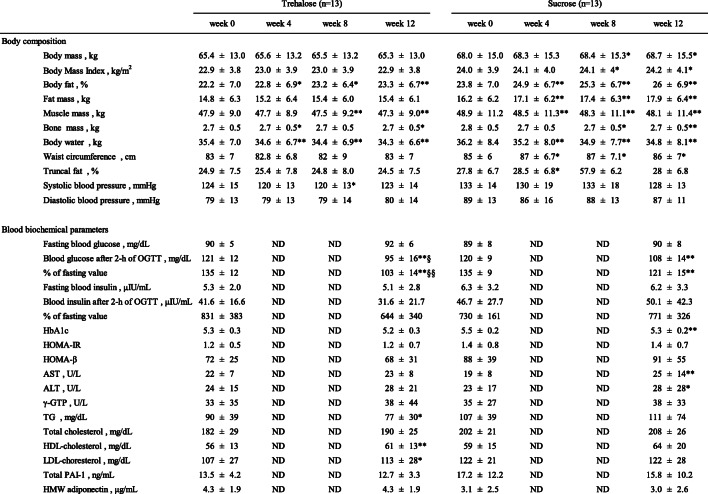
Body composition and blood biochemical parameters in participants with a postprandial blood glucose higher than the mean value

*HOMA-IR* homeostasis model assessment-insulin resistance; *HOMA-β* homeostasis model assessment-beta cell function; *AST* aspartate transaminase; *ALT* alanine transaminase; *γ-GTP* gamma-glutamyl transpeptidase; *TG* triglyceride; *PAI-1* plasminogen activator-inhibitor-1; *HMW adiponectin* high-molecular weight adiponectin

Data are expressed as mean ± SD (*n* = 13). Comparisons between two groups in the same week were made using the Mann-Whitney U-test. *P* values: ^§^*p* < 0.05, ^§§^*p* < 0.01. Comparisons between week 0 values and week 4, 8, and 12 values were made using the Wilcoxon signed-rank test. *P* values: *p < 0.05, **p < 0.01

### Analysis of the relationships between 2 h-PG values and other parameters at baseline

We also evaluated the relationships between 2-h PG, a measure of glucose tolerance, and the other parameters measured at baseline (Table 4). Univariate analyses showed that several parameters at baseline significantly correlated with baseline 2-h PG. WC and fat mass, which reflect obesity, yielded relatively high correlation coefficients. Furthermore, parameters that are closely related to glycemic control also showed close correlations, including plasma insulin concentration.

**Table 4:**
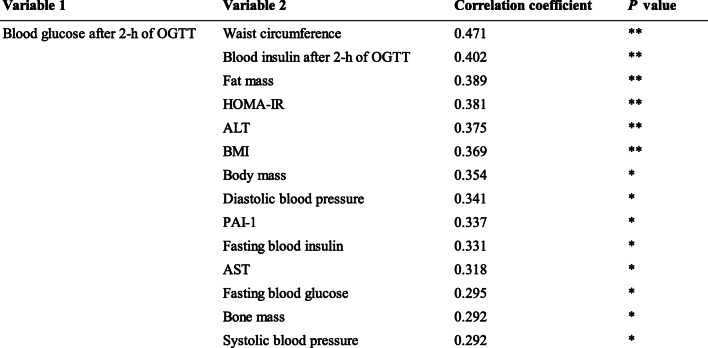
Correlations between changes in blood glucose after 2-h of an OGTT and body composition or blood biochemical parameters in the complete group of participants at baseline (*n* = 50)

*HOMA-IR* homeostasis model assessment-insulin resistance; *ALT* alanine transaminase; *BMI* body mass index; *PAI-1* plasminogen activator-inhibitor-1; *AST* aspartate transaminase

Spearman’s rank correlation coefficients are quoted. *P* values: * < 0.05, ** < 0.01

## Discussion

We have demonstrated that daily consumption of 3.3 g of trehalose improves glucose tolerance in healthy humans with relatively high postprandial plasma glucose concentrations to a similar extent to 10 g of trehalose [7]. Although 2-h PG at baseline was significantly higher than FPG, no difference was observed between 2-h PG and FPG in the trehalose group after 12 weeks of consumption (Table 2, Fig. 1). Therefore, daily trehalose consumption appears to quickly lower 2-h PG.

Analysis of data from all the participants showed that after 12 weeks of daily consumption of the two substances, 2-h PG in the trehalose group tended to be lower than in the sucrose group, but this difference was not significant (Fig. 1). In the present study, the participants had normal glucose tolerance, so the differences between 2-h PG and FPG were small, and it may therefore have been difficult to identify any differences between the groups. Therefore, the data from a subset of participants whose 2-h PG/FPG% was higher than the mean for all the participants were analyzed, and we found that the 2-h PG of this subset of the trehalose group was significantly lower than that of the equivalent subset of the sucrose group. In addition, the 2-h PG at the end of the study period was not significantly different from the FPG in trehalose group, whereas in the sucrose group, the 2-h PG was significantly higher than the FPG. Therefore, daily consumption of 3.3 g trehalose appears to quickly lower postprandial blood glucose. It has been reported that 2-h PG also increases with age in non-diabetic humans, and there is a risk of subsequent disease [14]. Therefore, we believe that it is important not to raise postprandial blood glucose, even in humans with a normal range of 2-h PG levels.

The 2-h PG was the primary endpoint of the present study. High postprandial blood glucose concentration is a risk factor for arteriosclerosis [8, 9]. Atherosclerosis is the pathologic basis of cardiovascular and cerebrovascular diseases, and about 20 million people die from atherosclerotic diseases every year. Temelkova-Kurktschiev et al. have shown that 2-h PG is a significant independent determinant of intima-media thickness, as a marker of atherosclerosis, in participants who are at risk of diabetes, using a multivariate analysis that included a variety of atherosclerotic risk factors [10]. Therefore, functional food components that reduce 2-h PG, such as trehalose, may reduce the risk of atherosclerosis.

In addition, trehalose is known to induce transcriptional activation of macrophage autophagy and autophagy-lysosome biosynthesis [15–17], which are thought to reduce atherosclerosis. Kaplon et al. have shown that oral trehalose improves resistance artery endothelial function [18]. Furthermore, dysfunctional autophagy in vascular smooth muscle cells has been shown to promote the development of arteriosclerosis and aortic aneurysm, because of cell death and aging [16]. Therefore, trehalose may also improve arteriosclerosis through the activation of autophagy and an improvement in glucose tolerance.

To identify factors that might affect 2-h PG, we evaluated the relationships between 2-h PG and other parameters. We found that WC and body fat mass were relatively closely correlated with 2-h PG (Table 4). These findings are consistent with those of Feng et al., who also showed that the standardized regression coefficients for the relationship between WC and 2-h PG are relatively high, using multivariate regression models, and that WC is strongly associated with type 2 diabetes mellitus [19]. This suggests that 2-h PG is closely related to central adiposity. The results of this study newly showed that increased abdominal circumference could increase postprandial blood glucose, even in healthy individuals without type 2 diabetes.

We have previously shown that the addition of trehalose suppresses HFD-induced mesenteric adipocyte hypertrophy and ameliorates glucose intolerance in mice, without reducing fat mass [4, 5]. An improvement in glucose tolerance in the absence of a reduction in fat mass has also been shown by Matsuzaka et al. in mice deficient in *Elovl6*, the gene that encodes the elongase responsible for the conversion of palmitate to stearate: obesity-induced insulin resistance is ameliorated through modulation of hepatic metabolism, without a concurrent reduction in obesity [20]. The authors concluded that not only the amount of fat but also the characteristics of the lipids, such as fatty acid length and degree of unsaturation, are important determinants of energy metabolism, and thus lifestyle-related diseases [20]. We also found that the addition of trehalose ameliorates the HFD-induced reduction in plasma HMW-adiponectin and increase in PAI-1, consistent with an improvement in insulin sensitivity, in our previous study [4, 5]. However, in the present study and in previous studies of healthy human participants, plasma HMW-adiponectin and PAI-1 were not affected by trehalose consumption. This apparent discrepancy can probably be explained by the fact the animals were obese and glucose intolerant, whereas the human volunteers were healthy. Therefore, to confirm the involvement of changes in adipose characteristics in the mechanism of the improvement in glucose tolerance in humans may require studies to be conducted in prediabetic and type 2 diabetic patients.

A single dose of trehalose does not stimulate a rapid increase in blood glucose or the excessive secretion of insulin or gastric inhibitory polypeptide, which promote fat accumulation, in healthy humans [21]. Furthermore, we have recently shown that daily administration of trehalose to healthy mice consuming a standard diet induces an increase in the number of beige adipocytes, accompanied by a reduction in adipocyte hypertrophy, higher body temperature, and lower blood glucose [22]. Therefore, regular consumption of trehalose may reduce adipocyte size and induce qualitative changes in adipocytes, which may result in lower postprandial blood glucose concentration. Further studies should be conducted in individuals with impaired glucose tolerance to identify differences in the effects of trehalose and sucrose on adipose quality, to determine whether these might mediate beneficial effects of trehalose on glucose tolerance.

In the present study, changes that were considered to be seasonal variations between summer and winter were recognized in both groups. Body fat percentage and fat mass significantly increased; and muscle mass, bone mass, and body water content significantly decreased in both groups (Tables 2 and 3), consistent with previous reports [23, 24]. Therefore, the changes in these parameters are not considered to be due to consumption of the test substances. In addition, at the end of the study period, the 2-h PG in the sucrose group was lower than that at baseline. The reason for this slight improvement in glucose tolerance is unclear. Nevertheless, future studies may need to account for seasonal effects in the analysis of their outcomes.

The limitations of the present study were that (i) the participants were healthy volunteers and (ii) they were employees of Hayashibara Co. Ltd., the study sponsor. (i) Our assertion that trehalose might help reduce postprandial blood glucose-related illnesses, such as arteriosclerosis, by reducing postprandial blood glucose is made on the basis of the results of studies conducted in healthy volunteers and in previous animal studies. Therefore, it is necessary to verify whether trehalose also improves glucose metabolism in patients with pre-diabetes in the future, to be able to draw firmer conclusions regarding its effect on arteriosclerosis and the progression to diabetes. (ii) This study was randomized, double-blind, placebo-controlled, parallel-group trial. And the identity of the substance being consumed by each participant remained confidential until all the data had been finalized, and the participants were blinded throughout the trial. Although we believe that this trial could eliminate or minimize biases, future trials may need to be conducted at the Contract Research Organization to eliminate biases.

## Conclusions

In conclusion, we have confirmed that daily consumption of trehalose improves glucose tolerance in non-diabetic people with higher postprandial glucose levels within the normal range. Furthermore, even when only a third of the previously tested dose is consumed, glucose tolerance improves to the same extent. These results suggest that the daily consumption of a teaspoon of trehalose in a meal reduces postprandial hyperglycemia, and it may reduce potentially therefore the risk of associated complications. Therefore, trehalose may also represent a useful food ingredient for people with pre-diabetes and postprandial hyperglycemia, to maintain health and improve quality of life.

## Data Availability

All data generated or analyzed during this study are included in the manuscript.

## Abbreviations

HOMA-IR: Homeostasis model assessment-insulin resistance
HOMA-β: Homeostasis model assessment-beta cell function
AST: Aspartate transaminase
ALT: Alanine transaminase
γ-GTP: Gamma-glutamyltransferase
TG: Triglyceride
PAI-1: Plasminogen activator-inhibitor-1
HMW adiponectin: High-molecular weight adiponectin
BMI: Body mass index
OGTT: Oral glucose tolerance test
2-h PG: 2-h plasma glucose during an OGTT
FPG: Fasting plasma glucose
